# Severe Acute Kidney Injury Associated with Transformation of Chronic Myelomonocytic Leukemia into Acute Myeloid Leukemia: A Case Report

**DOI:** 10.3390/jcm13020494

**Published:** 2024-01-16

**Authors:** Seong-Wook Lee, Mee-Seon Kim, Yong-Jin Kim, Hee-Yeon Jung, Ji-Young Choi, Jang-Hee Cho, Sun-Hee Park, Chan-Duck Kim, Yong-Lim Kim, Jeong-Hoon Lim

**Affiliations:** 1 Department of Internal Medicine, School of Medicine, Kyungpook National University, Kyungpook National University Hospital, Daegu 41944, Republic of Korea; maybee0914@gmail.com (S.-W.L.); hy-jung@knu.ac.kr (H.-Y.J.); jyss1002@hanmail.net (J.-Y.C.); jh-cho@knu.ac.kr (J.-H.C.); sh-park@knu.ac.kr (S.-H.P.); drcdkim@knu.ac.kr (C.-D.K.); ylkim@knu.ac.kr (Y.-L.K.); 2 Department of Pathology, School of Dentistry, Kyungpook National University, Kyungpook National University Hospital, Daegu 41944, Republic of Korea; meeseonkim@knu.ac.kr; 3 Department of Pathology, School of Medicine, Kyungpook National University, Kyungpook National University Hospital, Daegu 41944, Republic of Korea; yyjjkim@knu.ac.kr

**Keywords:** chronic myelomonocytic leukemia, acute myeloid leukemia, acute kidney injury, kidney biopsy, leukemic infiltration

## Abstract

Chronic myelomonocytic leukemia (CMML) is a rare hematologic disorder that infrequently causes acute kidney injury (AKI). CMML can transform into acute myeloid leukemia (AML), which can be accompanied by a deterioration in kidney function. However, severe AKI due to extramedullary manifestations of AML is rare. Herein, we present the case of a 67-year-old male patient with CMML that transformed into AML with severe AKI necessitating hemodialysis. The cause of the AKI was the AML transformation. The patient, with stable kidney function after chemotherapy for CMML, presented with a sudden decline in kidney function. Hemodialysis was initiated because of severe AKI, and histopathologic evaluation of the kidney biopsy specimen revealed severe, diffuse mixed inflammatory cell infiltrates in the interstitium and c-kit-immunopositive myeloblast-like cells. A bone marrow biopsy was performed because of the kidney biopsy findings suggesting that leukemic infiltration led to the diagnosis of AML. The patient received chemotherapy for AML, and his kidney function recovered. As illustrated in this case, severe AKI can develop as an early extramedullary manifestation during transformation from CMML to AML. Therefore, in patients with CMML and rapidly declining renal function, transformation into AML should be considered and histopathologically confirmed by kidney biopsy.

## 1. Introduction

Chronic myelomonocytic leukemia (CMML) is a rare, clonal hematopoietic malignancy with overlapping features of myeloproliferative neoplasm and myelodysplastic syndrome [1]. CMML can be categorized into three groups (CMML-0, CMML-1, and CMML-2) according to the percentage of blast cells in the bone marrow and peripheral blood [2]. CMML exhibits male predominance, with a median age of diagnosis in the seventh decade [3]. The estimated CMML incidence is 4 per 100,000 persons per year [4] and accounts for 11% of patients with myelodysplastic syndromes [5]. The clinical features of CMML are varied and include fatigue, general weakness, weight loss, drenching night sweats, and hepatosplenomegaly [6]. The extramedullary manifestations with CMML are diverse and can occur in certain organs including the lymph nodes, skin, gingiva, pleura, and pericardium [7,8,9,10]. These are associated with adverse outcomes [8,11]. In addition, kidney involvement in CMML is uncommon and can result from direct kidney infiltration [12,13]. Hypomethylating agents such as azacitidine are used to treat patients with CMML and can improve overall survival [14,15].

CMML can follow an aggressive course and transforms into secondary acute myeloid leukemia (AML) over 3–5 years in approximately 15% of all cases [16,17]. Patients diagnosed with AML typically present with constitutional symptoms or bone marrow failure, such as anemia, infections, and bleeding, although symptoms of extramedullary involvement can also occur [18]. In patients with AML, acute kidney injury (AKI) can develop for several reasons, including leukemic infiltration, medications, and ischemia [19]. AKI impacts the clinical course of AML and is a poor prognostic factor associated with increased mortality [19,20]. However, prompt diagnosis and appropriate treatment can facilitate rapid recovery of kidney function in patients with AML who develop AKI.

Herein, we present the case of a patient diagnosed with CMML who developed severe AKI, which was secondary to renal leukemic infiltration.

## 2. Case Presentation

The patient was a 67-year-old man who presented with symptoms of general weakness, mild peripheral edema in the lower extremities, and reduced urine output for four consecutive days. He was diagnosed with CMML-1 4 years before admission and received 37 cycles of azacitidine, administered every month from November 2019. Before the diagnosis of CMML, the patient was a former smoker (10 pack-years) and had quit 10 years ago. The patient occasionally used pain medications to manage spinal stenosis. Prior to the current admission, CMML was stable and kidney function was within the normal range, with serum creatinine levels of 1.02 mg/dL and 1.04 mg/dL one year and three months prior to admission, respectively. Laboratory tests at admission revealed a rapid decline in kidney function, leading to his referral to the Department of Nephrology. The patient denied taking medications with potential impact on kidney function, such as antibiotics, herbal supplements, or nonsteroidal anti-inflammatory drugs, before admission. Additionally, he had no history of hypovolemia, such as vomiting, diarrhea, or bleeding, and no chronic conditions, such as diabetes or hypertension. 

At admission, the patient’s blood pressure was 110/69 mmHg, pulse rate was 101 beats/min, and initial urine output was 660 mL/day. Oxygen saturation and body temperature were 97% and 36.6 °C, respectively. The general physical examination showed 2+ pitting edema in the legs and splenomegaly. 

Blood urea nitrogen and creatinine levels were 58.5 and 5.47 mg/dL, respectively, at admission. A complete blood count indicated a normal white blood cell count (6.48 × 10^9^/L) with anemia (hemoglobin, 6.9 g/dL) and mild thrombocytopenia (107 × 10^9^/L). A peripheral blood smear showed normocytic normochromic erythrocytes with a normal white blood cell count and relative monocytosis (51%). No blast cells or dysplastic cells were detected in the peripheral blood smear. The patient’s serum electrolyte levels were measured and found to be within normal ranges, except for a mild decrease in calcium levels (sodium, 130 mmol/L; potassium, 4.8 mmol/L; chloride, 106 mmol/L; bicarbonate, 15.7 mmol/L; calcium, 7.6 mg/dL; adjusted total calcium, 8.64 mg/dL; phosphate, 4.0 mg/dL; and uric acid, 8.6 mg/dL). The absence of hyperuricemia, hyperkalemia, and hyperphosphatemia ruled out the possibility of tumor lysis syndrome. Additionally, lactic acid and lactate dehydrogenase levels were measured and found to be within normal ranges (1.2 mmol/L (normal range: 0.7–2.5 mmol/L) and 376 U/L (normal range: 120–246 U/L), respectively). The creatine phosphokinase level was 48 U/L (normal range: 46–171 U/L), and the myoglobin level was 55.0 ng/mL (normal range: 0–154.9 ng/mL), both within the normal range. The C-reactive protein level was high (13.8 mg/dL), but there was no evidence of infection, and urine and blood cultures were negative.

Immunologic markers of glomerulonephritis, such as antinuclear, antineutrophil cytoplasmic, antiglomerular basement membrane, and antiphospholipase A2 receptor antibodies, were negative. Complement C3 and C4 levels were within the normal range. The patient was negative for hepatitis B and C and human immunodeficiency viruses, determined by serology, and serum cryoglobulin and protein electrophoresis were unremarkable. Urinalysis indicated 1+ proteinuria, with a urine protein/creatinine ratio of 2.21 g/g creatinine, and no microscopic hematuria. Nitrites, leukocyte esterase, and leukocytes were not detected in the urine. 

**Figure 1:**
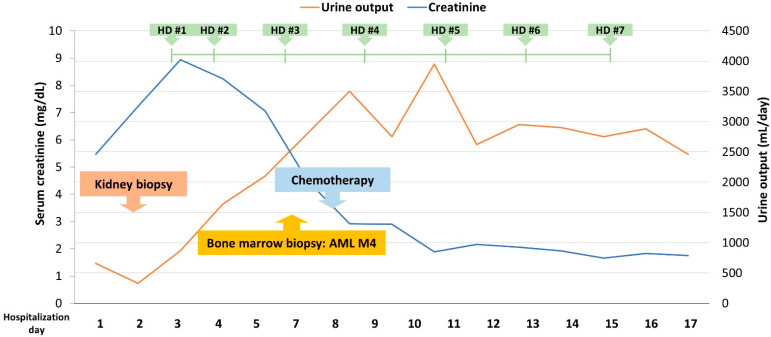
Summary of the clinical course. Abbreviations: HD, hemodialysis; AML, acute myeloid leukemia.

The patient underwent ultrasound-guided percutaneous kidney biopsy on the second day of hospitalization (Figure 1) and did not develop postprocedural complications. After hospitalization, the patient’s renal function continued to deteriorate, and his serum creatinine level increased to 8.94 mg/dL on hospitalization day 3. Chest radiography demonstrated bilateral infiltrates, and noncontrast-enhanced computed tomography of the abdomen revealed that both kidneys were normal in size and shape, without evidence of obstructive nephropathy. Despite the use of diuretics, follow-up chest radiography on day 3 of hospitalization revealed worsening pulmonary edema, which was accompanied by elevated serum creatinine levels and decreased urine output, and hemodialysis was initiated due to stable vital signs.

**Figure 2:**
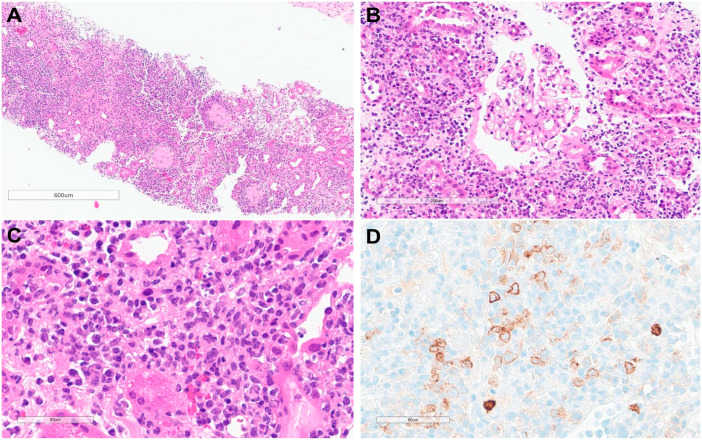
Histopathological findings of kidney biopsy. (**A**) Diffuse mixed inflammatory cells infiltrate in the interstitium with tubular atrophy. Additionally, 7 out of 10 glomeruli exhibited global sclerosis (Hematoxylin and Eosin; original magnification: ×40). (**B**) The glomerulus exhibits a ruptured Bowman’s capsule, accompanied by the infiltration of a few atypical cells into Bowman’s space (Hematoxylin and Eosin; original magnification: ×200). (**C**,**D**) Myeloblast-like cells are observed within a mixed inflammatory cell background (Hematoxylin and Eosin; original magnification: ×400), and the cells demonstrate a positive reaction to c-kit immunohistochemical staining (×400).

Light microscopic examination of the kidney biopsy specimen revealed global sclerosis in 7 of the 10 glomeruli (Figure 2A). Hematoxylin/eosin staining revealed diffuse mixed inflammatory cell infiltrates in the interstitium with severe tubular atrophy (Figure 2A). Normal-appearing glomeruli displayed Bowman’s capsule rupture, with a few atypical cells infiltrating Bowman’s space (Figure 2B). Furthermore, in areas with mixed inflammatory cell infiltrates, myeloblast-like cells were present; these cells were positive for c-kit and negative for CD34, determined by immunohistochemical staining (Figure 2C,D). Immunofluorescence staining revealed that the specimen was negative for immunoglobulin (Ig) G, IgA, IgM, C3, and fibrinogen. Electron microscopy revealed normal epithelial cells and glomerular basement membrane structures without electron-dense deposits. Based on the kidney biopsy results, AKI was considered as the main cause of leukemic infiltration. 

A bone marrow biopsy performed following hematology consultation confirmed the transformation of CMML into AML M4, with 21% blast cells. These blast cells were positive for MPO, CD13, CD14, CD33, CD38, CD45, CD64, and HLA-DR, and negative for CD3, CD5, CD7, CD10, CD19, CD20, and CD34. Chromosomal analysis of the bone marrow revealed a karyotype of 46, XY, +1, der(1;22)(q10;q10)[10]/45, XY, der(12;22)(q10;q10)[6]/46, XY[4]. Next-generation sequencing identified pathogenic mutations in the *Additional sex combs-like 1 (ASXL1)* and *Runt-related transcription factor 1 (RUNX1)* genes. Immunohistochemical staining of the bone marrow biopsy was positive for c-kit, CD45, and MPO.

The hematologist initiated less aggressive chemotherapy with low-dose cytarabine 20 mg/m^2^ subcutaneously, in combination with venetoclax 100 mg orally due to the patient’s poor Eastern Cooperative Oncology Group performance status and advanced age. Treatment for AML led to a reduction in serum creatinine level and the restoration of normal daily urine output, allowing for hemodialysis discontinuation on day 15 after hospitalization. At the three-month follow-up visit, the patient’s renal function was stable with a serum creatinine level of 1.5 mg/dL.

## 3. Discussion

In the present study, we report the rare case of a patient with CMML that transformed into AML, resulting in severe AKI. Hematologic malignancies can affect the kidneys, although kidney involvement is rare in CMML. The case presented here is a reminder of the potential sudden development of AKI in patients with CMML transforming into AML.

In a study of 825 patients with CMML, Morschhauser et al. reported that only 4 patients exhibited glomerulopathy with amyloidosis or extracapillary glomerulonephritis in the kidneys in the absence of leukemic cell infiltration [21]. Belliere et al. presented a case series aimed at refining the spectrum of kidney disorders associated with CMML [13]. The authors reported the renal histopathologic findings of eight CMML patients with AKI. Of these patients, four showed acute interstitial nephritis, two showed focal segmental glomerulosclerosis, and two showed chronic tubulointerstitial nephritis. The authors emphasized that renal complications of CMML are rare but associated with poor renal and global prognosis. Glomerulosclerosis, interstitial fibrosis, and tubular atrophy are the primary kidney lesions in these patients. CMML has an inherent risk of leukemic transformation, and various prognostic models have been proposed [16,22,23]. In one study of 274 patients with CMML, 36 (13%) patients experienced leukemic transformation, indicating an association between the presence of circulating blasts and the female sex [16]. AML typically presents with signs and symptoms of leukemic infiltration of bone marrow, such as pancytopenia. However, symptoms of extramedullary AML are also common. AML can affect multiple organs, including the kidneys, and can present as AKI [18].

AKI is frequently observed in patients with hematologic malignancies, particularly in those with AML. In one study, 36% of the patients with AML or high-risk myelodysplastic syndrome developed AKI [20]. They also reported that AKI was associated with an age of ≥55 years, mechanical ventilation, leukopenia, hypoalbuminemia, vasopressor use, and nephrotoxic agents such as vancomycin, amphotericin B, and diuretics [20]. In patients with hematologic malignancies, multiple mechanisms contribute to the development of AKI. In one study, Canet et al. reported that 68.5% of the patients experienced AKI and that hypoperfusion, tumor lysis syndrome, tubular necrosis, nephrotoxic agents, and hemophagocytic lymphohistiocytosis were the main causes, accounting for 91.4% of the AKI cases [19]. The range of AKI severity varies from asymptomatic to severe, rarely necessitating renal replacement therapy.

The patient presented here responded well to AML treatment, and his kidney function improved despite severe AKI requiring dialysis. However, AKI in patients with AML is associated with poor prognosis. A retrospective study of 401 patients with AML who underwent induction chemotherapy revealed an association between AKI and worse clinical outcomes [24]. In that study, 72 (18%) patients developed AKI during induction chemotherapy and more frequently required intensive care. Furthermore, the probability of achieving complete remission after chemotherapy is lower in these patients.

Kidney failure caused by the direct infiltration of leukemic cells is rare and has only been reported in 1% of all patients with acute leukemia [25]. The typical symptoms of renal infiltration are flank pain, abdominal distension, hematuria, and hypertension [26]. The present case is noteworthy because the transformation of CMML into AML was confirmed by the observation of renal infiltration and the characteristic histopathologic findings observed in the kidney biopsy specimen. Several studies have reported the clinical features of AKI due to leukemic infiltration requiring hemodialysis [27,28,29]. However, in these studies, patients with leukemia did not undergo kidney biopsy because of the high risk of bleeding, thereby hindering the confirmation of histologic findings in the kidneys. The present case emphasizes the importance of kidney biopsy in patients with AKI who are suspected to have undergone AML transformation [27,28].

Rapid deterioration of kidney function can manifest as an early clinical sign in patients with AML, as observed in the present case. Unlike the previously reported cases, the patient did not exhibit the typical symptoms of AML, such as pancytopenia, and the primary symptom was a rapid decline in kidney function requiring hemodialysis. The present case was unique because of the early onset of severe AKI during CMML transformation into AML, without other concurrent clinical manifestations. Histopathologic findings in kidney biopsy samples can be beneficial for the diagnosis of leukemic infiltration [30]. For example, tubular damage may be associated with acute tubular necrosis, glomerular infiltration indicates glomerulopathy, and interstitial infiltration can present as acute interstitial nephritis. Therefore, early AML diagnosis requires the identification of a correlation between AKI and leukemic infiltration. Immediate kidney biopsy should be performed in patients with CMML unexplained renal dysfunction for a confirmed diagnosis.

In patients with CMML, a rapid decline in kidney function in the absence of alternative causes might be due to the onset of AML and renal leukemic infiltration. Kidney biopsy can facilitate the definite diagnosis of AML, and early implementation of proper treatment can aid in managing leukemia and recovering kidney function.

## Data Availability

The original contributions presented in this study are included in the article, and further inquiries can be directed to the corresponding author.
